# Development of Nutrient-Rich Injera Using *Amaranthus* Leaf/Grain and Teff Flours: A Study on Nutritional Value, Sensory Characteristics, and Storage Stability

**DOI:** 10.1155/ijfo/2877941

**Published:** 2025-01-30

**Authors:** Betelhem Abera, Ramesh Duraisamy, Tewodros Birhanu, Beyene Gurbo, Ketemaw Salelign

**Affiliations:** ^1^Department of Chemistry (Industrial Chemistry Division), College of Natural and Computational Sciences, Arba Minch University, Arba Minch, Ethiopia; ^2^Department of Chemistry, College of Natural Sciences, Debark University, Debark, Ethiopia

**Keywords:** *Amaranthus*, flatbread, nutrition, sensory evaluation, shelf life, teff flour

## Abstract

Teff (*Eragrostis tef*, *Zucc*) is the smallest grain commonly cultivated in Ethiopia and Eritrea and is used mainly to prepare pancake-like staple food called flatbread (Injera). It has substantial national and global recognition due to its nutraceutical benefits and its gluten-free nature. Flatbread, a functional food for health promotion, can be enhanced with less mineral-rich crops like *Amaranthus*, which are often underutilized, to increase its nutritional and mineral content. Thus, the study is aimed at studying the nutritional, sensory, and storage period of flatbread by incorporating amaranth leaves and grain powders into teff flour. Experiments were designed (using Minitab software, v.19.1), and flatbread was prepared by altering the proportions (grams) of *Amaranthus* leaf and grain flour incorporated with teff flour in ratios of 2:98, 4:96, 6:94, 8:92, and 10:90 (*w*/*w*), teff being the significant component in each blend. The results revealed that most of the nutritive components (except carbohydrates and chromium (Cr)) improved significantly due to the incorporation of both (*Amaranthus* leaf and grains). Minerals such as sodium (Na), potassium (K), calcium (Ca), magnesium (Mg), iron (Fe), and zinc (Zn) increased noticeably (in percent), ranging from 10% to 12.2%, 0.02%–0.41%, 3%–4.4%, 0.33%–2.2%, 5.7%–10%, and 4.9%–28.7%, respectively. Furthermore, lower proportions of *Amaranthus* grain flour (2, 4, and 6 g blending) and leaf flour (2–10 g blending) significantly extended the storage period of blended flatbread up to 6–7 days, where higher proportions of *Amaranthus* grain (8 and 10 g blending) are expected to preserve the flatbread Injera up to 9–10 days, surpassing the 3–4 days of the control (100 g teff flour). Therefore, novel teff–*Amaranthus* blended food can be produced by means of a cost-effective and easy-to-produce new integrated teff–*Amaranthus* blended food that can enhance the nutritional value and shelf life of the existing teff Injera.

## 1. Introduction

Flatbread (Injera in the Amharic language) is a commonly consumed, fermented, traditional, and major staple food in Ethiopia and Eritrea. Nowadays, it is appreciated worldwide due to its gluten-free nature. It is a thin, pancake-like, spongy, circular flatbread with a small honeycomb-like structure on the top surface that is formed due to the production of CO_2_ during fermentation and escape during baking [1]. Flatbread is commonly prepared from teff powder, starter (ersho, a fluid saved from previously fermented dough), and water. Teff is the mainly accepted grain for the preparation of flatbread, although other grains (sorghum, maize, barley, wheat, and finger millet) are sometimes used [2].

This cereal is mainly preferred for making flatbread because it produces excellent products with desirable texture. However, teff could be a perfect dietary fiber, phenolic compound, and mineral source [3]. But this cereal-based flatbread can be preserved for up to 3 days at room temperature without compromising its organoleptic quality. So, there is a need to preserve (without diminishing the nutrition) the cereal-based flatbread using plant sources. In this context, underutilized *Amaranthus* grains/leaves are a choice to enhance the shelf life of the flatbread.

The plant *Amaranthus* (*Amaranthus cruentus*) largely served as good vegetarian food that appeared along with other main crops and was considered a weed. Additionally, the grain of *Amaranthus* was collected and served as local traditional medicine for allergies and amoebic dysentery. It has a huge biodiversity, and several of them are cultivated as leafy vegetables, grains, and ornamental plants, while many others are counted as weeds [4]. *Amaranthus* is also known for its high tolerance to poor soils; its resistance to drought, heat, and pests; and its ability to adapt to environments where conventional cereal crops do not grow well. *Amaranthus* can thus contribute to food security; particularly, it can serve as a good resource to poor communities [4].

*Amaranthus* leaf/grain provides an ideal amino acid composition for human nutrition. In particular, the content of lysine is high, and the remarkably higher contents of arginine and histidine make *Amaranthus* an interesting food source for child nutrition. Lysine content is considered the main reason for the higher protein quality of amaranth. The amino acid composition of *Amaranthus* protein can be compared to the WHO/FAO standard, 2016 [5]. In particular, *Amaranthus* grain is rich in calcium (Ca), magnesium (Mg), iron (Fe), potassium (K), and zinc (Zn). Due to its high nutritional value, it can be used in the same way as spinach; the leaf and grains of *Amaranthus* can even be preferably used to substitute/blend with other vegetables and cereals.

Escalating food prices and lower nutritional values severely affect any nation's food and nutritional security and macroeconomic stability. Many of the local vegetable varieties in Ethiopia are underexploited due to inadequate scientific knowledge of their nutritional potential [6, 7]. Although several studies have reported the compositional evaluation and functional properties of various edible wild plants in developing countries, much is still needed. *Amaranthus* (*Amaranthus cruentus*) is a widely available plant, but this plant is being ignored. However, previous research has found that this plant is very rich in minerals. Moreover, *Amaranthus* grain was served as a local traditional medicine for allergies and amoebic dysentery. Therefore, *Amaranthus* holds untapped potential in food production, serving as a valuable community resource to enhance food security and sustainability, as well as preserve food products, since it has valuable secondary metabolites like antioxidants [8, 9]. Hence, the primary aim of this research is to assess the nutritional benefits of incorporating *Amaranthus* leaves/grain and teff flour to prepare the Ethiopian flatbread (called Injera). The present study is mainly aimed at improving the nutritional value of existing traditional Ethiopian staple food called Injera, which is prepared from teff. Thus, this study promotes the scientific understanding of *Amaranthus* as a versatile source of both vegetables and grains in food preparation.

In this concern, the present study is aimed at developing newer blended food to enhance the nutritional value of food by blending the leaves and grains with teff flour. This blended flour was used to prepare the flatbread, and this was taken as the sample for the characterization in terms of nutritional and sensory analyses and shelf life evaluation.

## 2. Materials and Methods

### 2.1. Preparation of *Amaranthus* Leaf, Grain, and Teff Samples

*Amaranthus* leaves (2 kg) were thoroughly cleaned and were finely chopped and washed in cold water in a bowl, drained in the wash water, dried well in a hot air oven, and allowed to dry in shade for 3–5 days. The dried samples (approximately 1.5 kg) were then powdered and sieved using 500-*μ*m sieves and stored. The grains (2 kg) were prepared, sorted, washed, steeped, and germinated at room temperature. Then, the samples were dried carefully, and the rootlets were removed. The grains are then further dried and ground into flour (1.5 kg). Teff grain (2 kg) was ground into traditional flour fineness and stored in an airtight plastic bowl at room temperature for future analysis [10].

### 2.2. Experimental Design

This study used Minitab statistical software (Version 19.1, USA) for a constrained two-component mixture design for flatbread formulation. Different ratios of *Amaranthus* leaf/grain and teff are taken as independent variables (shown in Table 1). Proximate composition, mineral contents, pH, titratable acidity (TA), microbial load, and sensory evaluation were used as the dependent variables to test the significant differences between means of each sample in a treatment. The components were teff, *X*_1_ (in grams), and *Amaranthus* leaf/grain, *X*_2_ (in grams). The lower and upper bounds of the component variables were fixed based on the preliminary study conducted in the experimental site of the current study. The ratio of components is expressed as the mixture's ratio, and the total ratio (*X*_1_ + *X*_2_) is equal to 100 g. Teff flour (100 g) without the *Amaranthus* blending was taken as a control sample (C_0_).

### 2.3. Preparation of Blended Flatbread

As seen in the formulation in Table 1, accurately weighed teff flour was thoroughly mixed (using a laboratory blender) with different amounts of respective *Amaranthus* leaf/grain powders (used as fortifier). Blended teff and control flours (100 g) were mixed with water and starter culture (called ersho in Amharic) and then allowed to undergo primary fermentation for 48 h in a plastic bowl (capacity of 5 kg). The preparation of teff flatbread consists of two stages of natural fermentation, which is allowed 24–72 h, depending on ambient temperatures. About 10% of the fermented dough was mixed with water and boiled for 5 min to form an absit. It was added back to the batter and then allowed for a secondary fermentation for about 2–4 h. After fermentation, the dough was prepared and baked on fire for 4 min using an electric-powered hot clay pan to obtain the flatbread (flatbread). It was taken as a blended sample (AG_1_–AG_5_ and AL_1_–AL_5_), and the control sample (C_0_) had 100 g teff (without blending). The formulated blended and control flatbread was stored for proximate and sensory analyses, as well as for evaluating the shelf life and for characterization of nutritional value according to the earlier study [2]. Sensory evaluation was also carried out to test the acceptability of the final product.

### 2.4. Proximate Composition of Samples

The proximate composition (moisture, protein, fat, fiber, ash, and carbohydrates) of *Amaranthus* grains, leaves, teff, and the blended and control flatbread samples were determined according to the method of the Association of Official Analytical Chemists (AOAC), 2010 [11].

### 2.5. Estimation of Minerals

Graphite furnace atomic absorption spectroscopy techniques were used to analyze elements, including K, Ca, Mg, sodium (Na), Fe, Zn, chromium (Cr), and cadmium (Cd), with standard solutions prepared and analyzed in triplicate. The elemental composition of the sample (in milligrams/grams) was calculated [12]:

 $$\begin{array}{c} \text{Element }\left(\text{mg}/100\text{ g}\right)=\frac{\left(\text{sample concentration}\times \text{sample volume}\times \text{dilution factor}\right)}{\left(\text{sample weight}\right)}. \end{array}$$

### 2.6. Measurement of pH

An electronic pH meter (Jenway Model 370 pH/mV meter, England) was used to measure the pH of flatbread and raw materials. In this study, 5 g of the dried and powdered food sample was accurately weighed and was then dissolved in 50 mL of distilled water in a beaker, shaken well, and had its pH measured. The pH meter was calibrated before the measurement took place, using buffer solutions of pH 4 and 7.

### 2.7. Determination of Total TA

TA was determined by weighing about 5 g of powdered sample in a 100-mL flask containing 50 mL of distilled water and stirring well for 5 min. The supernatant liquid was separated, diluted to 10 mL, and titrated against NaOH (0.1 N) solution using phenolphthalein as an indicator until a pink color appeared [13]:

 $$\begin{array}{c} \text{Total titratable acidity }\left(\%\right)=\left[\left(N_{\text{NaOH}}\times V_{\text{NaOH}}\times M_{\text{w}}\right)/10\;S\right) \end{array}$$

where *M*_w_ is the molecular weight of lactic acid; *S* is the sample weight.

### 2.8. Sensory Evaluation

Semitrained panels, consisting of 50 panelists (men and women), were selected who regularly consume flatbread (Injera) as their staple food. A 7-point hedonic scale ranging from 1 to 7 was used. The standard hedonic evaluation format was used and evaluated the five attributes such as color, taste, texture, flavor, and overall appearance.

### 2.9. Rate of Mold Invasion

The flatbread sample was subjected to daily mold invasion monitoring for 8 days, with spoilage assessed by assessing the fungal invasion percentage on each roll. Measurement of mold outgrowth assessed on a flatbread roll was deemed positive if more than 1% of the total surface area was covered with fungi, which was considered spoilage according to the literature [14, 15].

### 2.10. Storage Period of the Prepared Flatbread Samples

Flatbread's storage period is the days it takes for mold growth to appear, while its microbial storage period refers to the days it takes for spoilage to occur. The flatbread surface samples were checked daily for mold appearance by the naked eye. The samples, both control and blended flatbread, were stored at room temperature in the laboratory and examined daily for any visible signs of the appearance of mold growth on the surface [15, 16].

### 2.11. Statistical Analysis

All experimental data were evaluated using statistical analysis using Minitab software (v.19.1, United States), and the results were expressed as mean and standard deviation (mean ± SD) of three replicates. The mean value was tested for significant differences by least significant difference (LSD), and the significance is accepted at *p* < 0.05.

## 3. Results and Discussion

### 3.1. Proximate and Mineral Analysis of Leaf and Grains of *Amaranthus*

The study analyzed the composition of powdered *Amaranthus* grains and leaf samples using standard methods. The results (Table 2) of the studied grain and leaf samples have shown a lower moisture content than literature values (30%–40% lower in leaf and grain has 0.5%). However, the leaf and grains of *Amaranthus* of the studied samples were observed to have lower moisture content than the WHO/FAO, 2016 [5] recommended as < 10%, which may restrict bacterial growth and enhance water retention and oil-repelling properties in baking goods.

As seen in the results in Table 2, the studied *Amaranthus* leaf and grains contain (in percentage) higher amount than literature values of carbohydrates (2%–8%), and other components include protein (76%–110% in leaf), fat (10 times high in leaf, 7%–14% in grain), fiber (7 times—7% in leaf, 1.4%–153%), and ash (42%–85% in leaf and 8% in grain). It reveals that the studied samples contain a good amount of all the nutrients needed for human consumption. Hence, the presently studied leaves and grains of *Amaranthus* are nutritious, with proximate compositions better than previous reports. This may be due to variations in factors such as climatic conditions, processing methods, soil quality, agroecology, age, maturity of the leaf/seed, and harvest season [15].

As seen in the results in Table 3, the study found that compared with the literature values, studied *Amaranthus* leaf has a high mineral content with Na, Mg, Zn, Fe (10%), and Ca (2.7%), but K is found to be 19% lower. In the case of *Amaranthus* grains, they show 5% of Ca and 2.6% of Fe higher, whereas other minerals Na, K, Mg, Zn, and Cr are lower as 5.4%, 46%, 13.5%, 7%, and 71%, respectively, than the literature value. In grains and leaves of *Amaranthus*, Cd is not reported.

The amounts of Zn (2.70 mg) and Cr (0.2 mg) are equal in leaf and grains. On the other hand, Cr content is found to be smaller than the literature value, and the absence of toxic heavy metal Cd in the samples makes the *Amaranthus* crop more promising and safer for human consumption, making it more promising for consumable goods.

### 3.2. Proximate Composition, pH, and Titratable Acid and Mineral Analysis of White Teff Flour and Raw Flatbread

#### 3.2.1. Proximate Composition, pH, and Titratable Acid

Generally, chemical compositions of cereals vary based on soil fertility, agroecology, maturity, cereal variety, fertilizer used, etc. Teff is important due to its attractive nutritional profile and gluten-free status, especially among gluten-sensitive patients. Comparative analyses of teff flour and flatbread were conducted and resulted in the composition (Table 4).

Teff flour, fermented and used in flatbread baking, has a unique taste and sourness, affecting microbe growth. It has larger carbohydrates and is a starchy crop, making it a potential gluten-free cereal. The study found a significant change in crude fat, fiber, ash content, carbohydrates, energy, pH, and titratable acid between teff flour and flatbread, whereas teff flour and flatbread had a closer amount of protein. In both cases, the moisture content was found to be lower (< 10%), which was recommended in the WHO/FAO standard, 2016 [5]. It ensures longer preservation and protection against microorganism growth. Also, teff flour and flatbread are more nutritious than cassava, indicating mineral availability varies based on climate and processing methods.

The results were compared with the literature; studied components such as carbohydrates, fat, ash, fiber, energy, and pH were found to be higher in teff flour at 29.8%, 50%, 40%, 1.08%, 31.6%, and 18%, respectively. However, some food components of teff flour were reported as lower: protein (1.9%), moisture (4.2%), and titratable acid (14.3%).

Similarly, in the case of the studied flatbread, it also had some changes compared with literature values, which are shown in Table 2. The percentage change was reported in a low amount of carbohydrates 2.07%, protein 10.4%, fiber 7.5%, moisture 16%, and titratable acid 4.6%. At the same time, 84%, 15.2%, and 2.4% high amounts of fat, ash, and energy were reported, respectively. These kinds of changes might be due to climatic conditions, cultivation location, soil quality, processing methods, etc.

#### 3.2.2. Mineral Analysis

Mineral analyses of studied teff flour and flatbread samples were compared with the literature values, and the results are presented in Table 5. Similar to proximate composition, results of minerals also, the study reported as that there is a significant change between the current study and literature values, as well as the teff flour and flatbread.

When compared to the earlier study, presently studied teff flour resulted in a low amount of K, Mg, Zn, and Fe, with 1.6%, 36.4%, 4.3%, 2.4%, and 43.4%, respectively. But, 85% of Na and 52.3% of Ca were reported (shown in Table 4). In the case of prepared flatbread, Mg and Zn were found to be high amounts at 6.7% and 39.4%, respectively; however, other minerals are lower as Na 13.6%, K 4.6%, Ca 10.7%, and Fe 9.3%. But, in both teff flour and flatbread, Cr is reported as 43.7% lower than the literature value. It confirms that the studied teff flour and its flatbread are safe and healthier for consumption.

### 3.3. Effect of *Amaranthus* on the Proximate Composition of Flatbread

The proximate composition and energy values of *Amaranthus* blended flatbread samples were analyzed, and the results were found to be significantly different compared to the control flatbread (shown in Table 6).

The moisture content of all blended flatbread samples varied between 5.20 ± 0.10 and 6.33 ± 0.20 g/100 g (shown in Table 6) than control flatbread, C_0_, which is 4.68 ± 0.02 g/100 g. However, the moisture content of all the studied formulations was found to be lower than 10% (Table 6), which is recommended by WHO/FAO guidelines (2016). Therefore, the presently studied *Amaranthus* leaf/grain blended teff flatbread was not susceptible to microbial spoilage. Thus, the studied formulations might provide a longer shelf life to store a long time.

The fiber content of *Amaranthus* grain–teff flatbread was significantly reduced compared to the control, whereas *Amaranthus* Leaves are the best blend materials for enhancing 1.2%–4.5% of fiber content. According to the WHO/FAO standard, 2016 [5], the recommended level of foodstuff fiber could be > 10%. Hence, currently studied raw materials and *Amaranthus* Grain/leaf-blended flatbread samples were considered better edible food formulations.

The crude protein in formulated flatbread was significantly influenced by the increment of the blending ratio of *Amaranthus* grains having higher protein content (range of increases 1.8%–19.5%). This could be due to the relatively higher protein content (12.8 ± 0.33 g) of *Amaranthus* grain. As seen in the results in Table 6, the blending ratio had a significant effect (*p* < 0.05) on the fat content of flatbread samples due to variations in ingredients. Comparatively, the *Amaranthus* Grain improves the fat content from 45.8% to 93.3% to reach the WHO/FAO, 2016 [5] recommended level of fat content (10%–15%). Thus, studied *Amaranthus* grain blended teff flatbread evidenced to fulfill the protein and fat for the human diet.

The ash content of *Amaranthus*–teff flatbread blends fluctuated significantly due to the formulation ratios, with the range of increment from 3.00 ± 0.30 (with 4.2% increase) to 5.60 ± 0.18 (94.4% increase). Also, according to the WHO/FAO standard, 2016 [5], the ash content of the currently studied composite flour-baked flatbread (flatbread) agrees with the recommended level (3%–5%). Hence, this study reveals that the blending of *Amaranthus* leaf and grain flour might be a better alternative food crop that can be used to blend into the teff flour for further processing to bake the flatbread. The total energy of *Amaranthus* grain flatbread resulted in the current study range between 357.9 ± 0.03 and 382.4 ± 0.35 kcal (Table 6).

This is found to be above the minimum requirement of energy (350 kcal/100 g) reported in the WHO/FAO standard, 2016 [5]. Hence, the energy values of the presently studied samples show that the grain and leaf portions of the *Amaranthus* crop are better alternate materials to fortify any cereals like teff. The pH and titratable acid levels do not show statistical significance by blending of teff with *Amaranthus* grain and leaf flour (as shown in Table 6).

The edible carbohydrate content (excluding the fiber) in blends was lowered with a significant effect (*p* < 0.05) from 61.80 ± 0.31 to 69.42 ± 0.10 g (Table 6) with decrements ranging from 1.9% to 12.7%, respectively. Degradation and a subsequent decrease in starch and soluble sugars are expected to occur during the fermentation of teff flour during flatbread preparation. However, this reduction in carbohydrate content may not influence much the nutritional value of flatbread. The recommended carbohydrate level, as per WHO/FAO, 2016 [5], is 60%–75%. Accordingly, all the *Amaranthus* grain/leaf blended teff flatbread is recommended to be consumed to fulfill the need for carbohydrates for consumers.

### 3.4. Effect of Blending of *Amaranthus* on Sensory Evaluation of Flatbread

A panel of 50 experts evaluated flatbread prepared with different proportions of *Amaranthus* grain/leaf and teff compared to the control flatbread. Results have shown significant differences (*p* < 0.05) in sensory attributes, possibly due to blending proportions of composite flours. However, most of the proportional flatbread formulations were liked in an acceptance level closer to the control. The color reduction in flatbread can be attributed to fermentation and processing, requiring careful attention. The taste of flatbread is associated with sweet, sour, and bitter sensations (Table 7).

Flatbread, made from teff and *Amaranthus* composite flours, has a slightly reduced taste and texture when teff level is reduced. However, adding more *Amaranthus* grain or leaf flour moderately reduces the quality. The overall acceptability of formulated flatbread is similar (in AG_1_ and AG_2_) and closer to the control (native flatbread has 100% teff) in many cases except AL_5_ formulation. Thus, *Amaranthus* leaf and grains were found to be a better alternative for the blending of flatbread in terms of sensory evaluation score.

### 3.5. Effect of *Amaranthus* on the Mineral Composition of Flatbread

Mineral contents of the *Amaranthus* blended baked flatbreads were measured and are described in Table 8. It has been clearly revealed that the composition of minerals increased significantly (*p* < 0.05) as the levels of blending increased. This could be described as increased levels of Na (10%–12.2%), K (0.02%–0.41%), Ca (3%–4.4%), Mg (0.33%–2.2%), Fe (5.7%–10%), and Zn (4.9%–28.7%) as a result of blending the *Amaranthus* grains and leaves. The increment of Na, Fe, and Zn is appreciable than other minerals. This may be due to the richness of these minerals in teff flour and *Amaranthus* crops. Furthermore, the amount of Cr in *Amaranthus* grain/leaf blended flatbread was showed to be nonsignificant with a lower amount including the control sample. It seems to be *Amaranthus* blended and unblended flatbreads are safe to consume.

The maximum increment, 28.7% of Zn (2.87 ± 0.10 mg/100 g in AG_5_ sample), was noticed in *Amaranthus* grain blended flatbread. This may help to increase the consumption of *Amaranthus* crops. Generally, increased consumption of vegetables is critical to reducing the incidence of nutritional deficiencies of elements such as Zn, which is essential for carbohydrate metabolism and protein synthesis. Zn participates in all major biochemical pathways [17]. Diets like moringa leaf powder, which is rich in micronutrients, are strongly recommended by Ryan et al. [15]. Accordingly, *Amaranthus* leaf and grain parts are also recommended for consumption to reduce nutritional deficiency. Thus, the present investigation shows that the *Amaranthus* crop has an adequate amount of minerals, and it has been suggested that the leaf and grains of *Amaranthus* can be used as an alternative crop to enrich the mineral contents of any cereals by means of blending.

### 3.6. Evaluation of Storage Period by Mold Invasion Rate of Control and Blended Flatbreads

The shelf life of flatbread is defined as the period (in days) when mold growth can be observed first by the naked eye. Mold growth on the flatbread is an indicator of its length of shelf life. Baked flatbread samples (control and fortified flatbread) were carefully stored at room temperature. The shelf life of a sample is expressed in relation to the corresponding control [2, 14].

**Figure 1:**
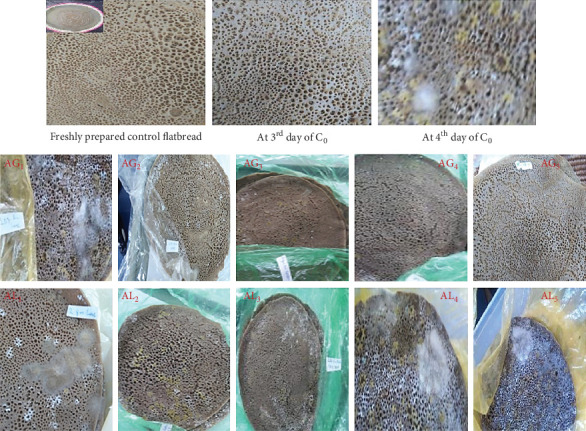
Observation of mold growth in control flatbread (at 1–4 days of storage) and *Amaranthus* grain (AG_1_–AG_5_) and leaf (AL_1_–AL_5_) blended flatbreads on the eighth day of storage.

Newly prepared flatbreads are free of fungus, but owing to its larger surface, there is a chance of it getting spoiled by fungi. Temperature is one of the most important environmental factors that affect the development and propagation of spoilage molds in vitro as well as in vivo [14]. The measurement of the mold outgrowth was observed and is presented in Figure 1.

In this current study, the spoilage of flatbread was evaluated based on the percentage of total surface area of each roll covered by fungal growth. Mold invasion on control and *Amaranthus* grain/leaf blended flatbread samples was monitored until the sample surface area spoiled by 1% [14, 15].

The study examined the growth rates of molds; accordingly, control flatbread (C_0_) showed white fungal colonies, which were dominated by grown microbes within 4 days of storage. In contrast, the blended flatbreads with lower proportions of *Amaranthus* grain (AG_1_–AG_3_) flour and all the studied proportions (AL_1_–AL_5_) of leaf flour took a similar longer time (up to 8 days) to notify spoilage. A higher amount of grain flour blended samples was found to be better for withstanding mold attacks, but all samples deteriorated on the eighth day (except AG_4_ and AG_5_, shown in Figure 1). The study suggests that the *Amaranthus* plant contains antioxidants, potentially aiding in prolonged storage.

Hence, blended flatbreads (with lower proportions of grain and all studied proportions of leaf) were considered safe until the sixth day; but the higher amount (8 and 10 g) of *Amaranthus* blending showed better for consumption, still expected it may protect (preserve) the samples for longer time (probably 9–10 days).

**Figure 2:**
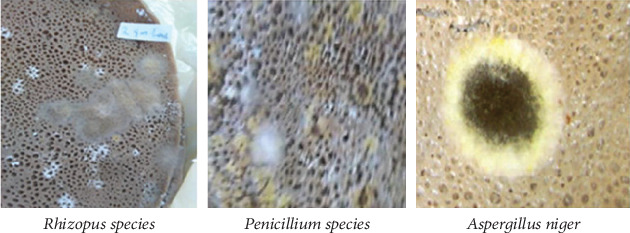
Observation of the different kinds mold growth on fortified flatbread on the eighth day at 28.5°C.

Extensive growth of molds was observed starting from the seventh day, and a complete invasion was noticed in many cases on the eighth day of storage (as shown in Figure 1) with three kinds of molds. Its production on the flatbread was isolated at ambient temperature and detected by microscopic evaluation (given in Figure 2). After perceiving the morphological features of the colony, it was found that these molds belong to *Rhizopus* spp., *Penicillium* spp., and *Aspergillus niger*. *Rhizopus* is a white to dark gray fungus that is known as common bread mold and is usually observed on aged flatbreads. As stated by Gock et al. [17] and Ashagrie and Abate [2], *Rhizopus* is a nonseptate mycelium with root-like rhizoids and black columellate, having sporangiophores in clusters. *Penicillium* species are xerophilic fungi that frequently bring about spoilage of baked foods. *Aspergillus niger* black colonies become more dominant and are visible to the naked eye when the temperature becomes warmer than ambient.

## 4. Conclusions

The study found that *Amaranthus* grain and leaf flours blended with the teff flour can significantly enhance the nutrients of teff flatbread as 19.5% of protein, fat (range: 45.8%–93.3%), ash (4.2%–94.4%), energy (2.3%–9.6%), Na (10%–12.2%), Fe (5.7%–10%), and Zn (4.9%–28.7%), and other minerals such as K, Mg, and Ca were also significantly increased by the increment of blending the grains/leaves of *Amaranthus*. In the case of carbohydrates, it declined from 1.9% to 12.7% while increasing the blending of teff. But mostly, the nutrients are closer in agreement with the WHO/FAO recommended level. Formulated flatbread samples, made from teff flour and *Amaranthus* grain and leaf flour, showed positive sensory attributes comparable to traditional flatbread.

The study reveals the shelf life of the control (100 g teff flatbread) was 4 days of storage stability; all cases of *Amaranthus* leaf blended (AL_1_–AL_5_) and up to middle-level *Amaranthus* grain blended teff flatbreads (AG_1_–AG_3_) reported the shelf life 6–7 days of storage stability for consumption, whereas samples AG_4_ and AG_5_ are expected to have 9–10 days of storage stability, which is confirmed through mold invasion study of the studied samples.

Thus, the study concludes that *Amaranthus* grain/leaf flours could be promising alternative blending materials to the flatbread and other food items to enrich the nutrients and shelf life. However, further studies are needed to evaluate its health benefits and grain productivity.

## Figures and Tables

**Table 1:** Blending proportions of *Amaranthus* grain flour and *Amaranthus* leaves flour with teff flour and its sample code.

**Mixing of teff and *Amaranthus* grain (AG) flour**			**Blending of teff and *Amaranthus* leaf (AL) flour**		
**Sample code**	**Amount of teff flour (g)**	**Amount of AG (g)**	**Sample code**	**Amount of teff flour (g)**	**Amount of AL (g)**
AG_1_	98	2	AL_1_	98	2
AG_2_	96	4	AL_2_	96	4
AG_3_	94	6	AL_3_	94	6
AG_4_	92	8	AL_4_	92	8
AG_5_	90	10	AL_5_	90	10
Control (C_0_)	100	0		100	0

Abbreviations: AG, *Amaranthus* grain; AL, *Amaranthus* leaf.

**Table 2:** Proximate composition, pH, and titratable acidity of *Amaranthus* leaf and grains.

**Parameters (per 100** g**)**	**Leaf powder**		**Grain powder**	
	**Current study**	**Literature value**	**Current study**	**Literature value**
Moisture (g)	9.7 ± 0.04^a^	16.9–11.7	9.6 ± 0.05^b^	9.8
Fiber (g)	7.5 ± 0.34^a^	1.3–7.0	3.30 ± 0.20^b^	1.3–2.9
Fat (g)	5.1 ± 0.51^b^	0.3–0.5	8.0 ± 0.32^a^	7.0–7.5
Protein (g)	7.4 ± 0.02^b^	3.5–4.2	12.8 ± 0.33^a^	14–15.0
Ash (g)	3.7 ± 0.31^a^	2–2.6	2.6 ± 0.19^b^	2.4–2.6
Carbohydrates (g)	70.8 ± 0.29^a^	65–68.4	68.2 ± 0.44^b^	63–65
Energy (kcal/100 g)	358.7 ± 0.41^a^	350–360	356.0 ± 0.21^b^	371–391
Titratable acidity	2.1 ± 0.72^a^	2.2	1.9 ± 0.38^b^	2.1
pH	6.9 ± 0.04^a^	7.0	7.03 ± 0.21^a^	6.98

*Note:* The superscripts of the current study results between grain and leaf (in rows) that share different alphabets indicate statistical significance (*p* < 0.05).

**Table 3:** Mineral composition of *Amaranthus* leaf and grains.

**Mineral content (mg/100 g)**	**Leaf powder**		**Grain powder**	
	**Current study**	**Literature value**	**Current study**	**Literature value**
Na	22.73 ± 0.28^a^	—	22.30 ± 0.33^b^	23.5
K	344.80 ± 1.02^b^	411	347.80 ± 0.10^a^	508.0
Ca	267 ± 0.93^a^	260	157.50 ± 0.33^b^	150.0
Mg	169 ± 2.10^b^	—	218.50 ± 0.49^a^	248.0
Zn	2.70 ± 0.99^a^	—	2.70 ± 0.21^a^	2.90
Fe	4.30 ± 2.19^b^	3.90	7.80 ± 0.28^a^	7.60
Cr	0.2 ± 3.83^a^	—	0.2 ± 0.40^a^	0.70
Cd	NR	—	NR	—

*Note:* The superscripts of the current study results between grain and leaf (in rows) that share different alphabets indicate statistical significance (*p* < 0.05).

Abbreviation: NR, not reported.

**Table 4:** Results of proximate composition, pH, and titratable acidity and flatbread of the current study with the literature values.

**Parameters (g/100** g**), dry weight basis**	**Teff flour**		**Prepared flatbread**	
	**Current study result**	**Literature value**	**Current study result**	**Literature value**
Carbohydrates	74.80 ± 0.21^a^	57	70.80 ± 0.22^b^	72.30
Protein	10.20 ± 0.03^a^	10.40	10.80 ± 0.10^a^	12.05
Fiber	4.10 ± 0.02^b^	3.7	6.20 ± 0.32^a^	6.70
Fat	3.60 ± 0.10^b^	2.4	4.80 ± 0.41^a^	2.61
Ash	3.50 ± 0.21^a^	2.50	2.88 ± 0.03^b^	2.5
Moisture (%)	3.60 ± 0.03^b^	3.76	4.68 ± 0.21^a^	5.6
Energy (kcal)	383.2 ± 0.21^a^	291.2	369.6 ± 0.02^b^	360.89
pH	3.20 ± 0.31^b^	2.71	3.40 ± 0.08^a^	3.40
Titratable acidity	1.80 ± 0.04^b^	2.10	2.80 ± 0.02^a^	2.78

*Note:* Comparison of the results of studied teff and flatbread in each row which shared different superscripts, indicating statistical significance (*p* < 0.05).

**Table 5:** The results of mineral compositions of Teff flour and prepared flatbread (Injera) of the current study with literature values.

**Mineral content (mg/100 g)**	**Teff flour**		**Prepared flatbread**	
	**Current study result**	**Literature value**	**Current study result**	**Literature value**
Na	12.20 ± 0.09^b^	6.60	24.2 ± 0.32^a^	28.01
K	343.80 ± 0.21^a^	349.3	340.5 ± 0.19^b^	357.04
Ca	120.0 ± 0.33^a^	78.80	70.40 ± 0.32^b^	78.80
Mg	108.20 ± 0.41^b^	170	120.8 ± 0.16^a^	113.2
Zn	2.20 ± 0.29^a^	2.30	2.23 ± 0.21^a^	1.6
Fe	9.62 ± 0.04^b^	9.86	9.80 ± 0.22^a^	10.81
Cr	0.40 ± 0.39^a^	0.71	0.40 ± 0.02^a^	0.71
Cd	Not reported	0.03	Not reported	—

*Note:* Comparison of the results of studied teff and flatbread in each row which shared different superscripts, indicating statistical significance (*p* < 0.05).

**Table 6:** Proximate composition, pH, and titratable acidity value of *Amaranthus* grain/leaf blended and control flatbreads.

**Proximate components**	**Results of proximate composition obtained**											
	**C**_**0**_	**AG**_**1**_	**AG**_**2**_	**AG**_**3**_	**AG**_**4**_	**AG**_**5**_	**C**_**0**_	**AL**_**1**_	**AL**_**2**_	**AL**_**3**_	**AL**_**4**_	**AL**_**5**_
Moisture (g)	4.68 ± 0.21^c^	6.20 ± 0.01^c^	6.28 ± 0.19^b^	6.32 ± 0.02^a^	6.31 ± 0.21^a^	6.33 ± 0.20^a^	4.68 ± 0.21^d^	5.20 ± 0.10^c^	5.30 ± 0.21^b^	5.30 ± 0.21^b^	5.38 ± 0.22^a^	5.41 ± 0.22^a^
Carbohydrates (g)	70.80 ± 0.22^a^	64.22 ± 0.2^b^	63.58 ± 0.21^c^	62.98 ± 0.31^d^	62.66 ± 0.04^d^	61.80 ± 0.31^e^	70.8 ± 0.22^a^	67.10 ± 0.21^e^	67.90 ± 0.23^d^	68.90 ± 0.20^c^	69.12 ± 0.21^b^	69.42 ± 0.10^a^
Fat (g)	4.80 ± 0.41^f^	7.00 ± 0.21^e^	8.03 ± 0.19^d^	8.26 ± 0.21^c^	8.76 ± 0.07^b^	9.28 ± 0.32^a^	4.80 ± 0.41^e^	4.83 ± 0.22^e^	5.02 ± 0.32^d^	5.16 ± 0.11^c^	5.24 ± 0.32^a^	5.26 ± 0.05^a^
Fiber (g)	6.20 ± 0.32^a^	5.02 ± 0.20^c^	5.05 ± 0.16^c^	5.08 ± 0.21^c^	5.12 ± 0.26^b^	5.17 ± 0.18^b^	6.20 ± 0.32^c^	6.28 ± 0.30^c^	6.36 ± 0.20^b^	6.38 ± 0.32^b^	6.40 ± 0.21^a^	6.48 ± 0.05^a^
Protein (g)	10.80 ± 0.10^e^	12.2 ± 0.02^d^	12.35 ± 0.07^c^	12.56 ± 0.17^b^	12.87 ± 0.19^a^	12.91 ± 0.19^a^	10.80 ± 0.10^d^	11.50 ± 0.31^a^	11.35 ± 0.42^b^	11.0 ± 0.39^c^	10.64 ± 0.42^e^	10.68 ± 0.20^e^
Ash (g)	2.88 ± 0.03^e^	4.10 ± 0.01^a^	3.40 ± 0.15^b^	3.50 ± 0.21^b^	3.20 ± 0.39^c^	3.00 ± 0.30^d^	2.88 ± 0.03^f^	3.10 ± 0.31^e^	3.50 ± 0.31^d^	4.00 ± 0.02^c^	4.70 ± 0.03^b^	5.60 ± 0.18^a^
Energy (kcal)	369.6 ± 0.02^e^	368.9 ± 0.02^d^	375.9 ± 0.08^c^	376.5 ± 0.39^c^	380.9 ± 0.71^b^	382.4 ± 0.35^a^	369.6 ± 0.02^a^	357.9 ± 0.03^e^	362.2 ± 0.00^d^	366.4 ± 0.10^c^	366.2 ± 0.43^c^	367.7 ± 0.32^b^
Titratable acidity	2.80 ± 0.02^a^	2.74 ± 0.03^a^	2.74 ± 0.17^a^	2.78 ± 0.21^a^	2.82 ± 0.71^a^	2.83 ± 0.21^a^	2.80 ± 0.02^a^	2.80 ± 0.31^a^	2.81 ± 0.32^a^	2.83 ± 0.32^a^	2.87 ± 0.10^a^	2.88 ± 0.32^a^
pH	3.40 ± 0.08^a^	3.84 ± 0.20^a^	3.82 ± 0.13^e^	3.85 ± 0.39^a^	3.87 ± 0.41^a^	3.88 ± 0.18^a^	3.40 ± 0.08^a^	4.00 ± 0.22^a^	3.94 ± 0.20^a^	3.91 ± 0.39^a^	3.87 ± 0.21^a^	3.82 ± 0.17^a^

*Note:* Data in each row (with respect to grains and leaf along with control) sharing different letters of superscript are statistically significant (*p* < 0.05).

Abbreviations: AG, *Amaranthus* grain; AL, *Amaranthus* leaf.

**Table 7:** Sensory evaluation score for teff–*Amaranthus* flatbread given by panelists.

**Sample code**	**Color**	**Taste**	**Texture**	**Flavor**	**Overall acceptability**
C_0_ (control)	7.00 ± 0.05^a^	6.98 ± 0.23^a^	6.96 ± 0.09^a^	6.97 ± 0.05^a^	7.00 ± 0.30^a^
AG_1_	6.94 ± 0.16^a^	6.90 ± 0.15^a^	6.92 ± 0.23^a^	6.95 ± 0.60^a^	6.98 ± 0.15^a^
AG_2_	6.90 ± 0.82^a^	6.88 ± 0.06^a^	6.90 ± 0.18^a^	6.91 ± 0.25^a^	6.95 ± 0.09^a^
AG_3_	6.53 ± 0.21^b^	6.52 ± 0.16^b^	6.21 ± 0.22^c^	6.50 ± 0.31^b^	6.48 ± 0.33^b^
AG_4_	6.29 ± 0.14^c^	6.38 ± 0.03^c^	6.50 ± 0.42^b^	6.14 ± 0.22^c^	6.31 ± 0.12^c^
AG_5_	6.21 ± 0.22^b^	6.29 ± 0.18^c^	6.18 ± 0.09^c^	6.09 ± 0.49^c^	6.28 ± 0.07^c^
C_0_ (control)	7.00 ± 0.05^a^	6.98 ± 0.23^a^	6.96 ± 0.09^a^	6.97 ± 0.05^a^	7.00 ± 0.30^a^
AL_1_	6.50 ± 0.01^b^	6.55 ± 0.23^b^	6.83 ± 0.01^a^	6.58 ± 0.24^b^	6.69 ± 0.15^b^
AL_2_	6.00 ± 0.22^c^	6.36 ± 0.04^c^	6.64 ± 0.11^b^	6.52 ± 0.12^b^	6.55 ± 0.04^c^
AL_3_	6.32 ± 0.43^c^	6.31 ± 0.19^c^	6.61 ± 0.25^b^	6.27 ± 0.23^c^	6.17 ± 0.18^d^
AL_4_	5.72 ± 0.33^d^	6.55 ± 0.24^b^	6.57 ± 0.10^b^	6.23 ± 0.08^c^	6.11 ± 0.21^d^
AL_5_	5.63 ± 0.07^d^	6.29 ± 0.08^c^	6.10 ± 0.08^c^	6.20 ± 0.13^c^	5.90 ± 0.05^e^

*Note:* Data in column sharing different letters of superscript are statistically significant (*p* < 0.05).

Abbreviations: AG, *Amaranthus* grain; AL, *Amaranthus* leaf.

**Table 8:** Mineral composition of *Amaranthus* grain/leaf blended and unblended control flatbread.

**Mineral**	**Results of mineral contents obtained (mg/100** g**)**											
	**C**_**0**_	**AG**_**1**_	**AG**_**2**_	**AG**_**3**_	**AG**_**4**_	**AG**_**5**_	**C**_**0**_	**AL**_**1**_	**AL**_**2**_	**AL**_**3**_	**AL**_**4**_	**AL**_**5**_
Na	24.20 ± 0.32^e^	26.68 ± 0.01^d^	26.81 ± 0.40^c^	26.97 ± 0.30^b^	27.18 ± 0.2^a^	26.20 ± 0.20^f^	24.20 ± 0.32^d^	26.43 ± 0.25^d^	26.64 ± 0.27^c^	26.73 ± 0.41^c^	26.87 ± 0.21^b^	27.03 ± 0.11^a^
K	340.5 ± 0.19^d^	340.7 ± 0.30^c^	340.8 ± 0.40^c^	341.1 ± 0.10^b^	341.6 ± 0.1^a^	341.90 ± 0.20^a^	340.5 ± 0.19^b^	340.6 ± 1.21^b^	340.7 ± 0.35^b^	340.8 ± 0.53^b^	340.9 ± 0.36^a^	341.2 ± 0.33^a^
Mg	120.8 ± 0.16^d^	122.2 ± 0.40^c^	122.3 ± 0.20^c^	122.7 ± 0.20^b^	123.1 ± 0.3^a^	123.40 ± 0.20^a^	120.8 ± 0.16^c^	121.2 ± 0.20^b^	121.3 ± 0.12^b^	121.5 ± 0.40^b^	121.9 ± 0.31^a^	122.1 ± 0.46^a^
Ca	70.4 ± 0.32^d^	72.55 ± 0.02^c^	72.78 ± 0.30^c^	72.97 ± 0.20^b^	73.27 ± 0.0^a^	73.40 ± 0.90^a^	70.4 ± 0.32^c^	72.60 ± 0.31^b^	72.80 ± 0.76^b^	73.00 ± 0.24^b^	73.30 ± 0.14^a^	73.50 ± 0.41^a^
Fe	9.80 ± 0.22^a^	10.78 ± 0.07^a^	10.75 ± 0.30^a^	10.71 ± 0.10^b^	10.65 ± 0.1^c^	10.52 ± 0.90^d^	9.80 ± 0.22^a^	10.60 ± 0.70^b^	10.47 ± 1.2^c^	10.40 ± 0.12^d^	10.37 ± 0.21^d^	10.36 ± 1.00^d^
Zn	2.23 ± 0.21^d^	2.32 ± 0.30^d^	2.28 ± 0.10^d^	2.52 ± 0.20^c^	2.67 ± 0.30^b^	2.87 ± 0.10^a^	2.23 ± 0.21^c^	2.27 ± 0.35^d^	2.34 ± 0.62^c^	2.40 ± 0.35^b^	2.47 ± 0.16^b^	2.79 ± 0.11^a^
Cr	0.40 ± 0.02^a^	0.32 ± 0.04^a^	0.34 ± 0.20^a^	0.36 ± 0.03^a^	0.35 ± 0.20^a^	0.38 ± 0.10^a^	0.40 ± 0.02^a^	0.30 ± 0.33^a^	0.33 ± 0.04^a^	0.33 ± 1.32^a^	0.37 ± 0.38^a^	0.39 ± 0.32^a^

*Note:* Data in each row (with respect to grains and leaf along with control) sharing different letters of superscript are statistically significant (*p* < 0.05).

Abbreviations: AG, *Amaranthus* grain; AL, *Amaranthus* leaf.

## Data Availability

The data that support the findings of this study are available from the corresponding author upon reasonable request.
